# Adolescents' Neural Processing of Risky Decisions: Effects of Sex and Behavioral Disinhibition

**DOI:** 10.1371/journal.pone.0132322

**Published:** 2015-07-15

**Authors:** Thomas J. Crowley, Manish S. Dalwani, Susan K. Mikulich-Gilbertson, Susan E. Young, Joseph T. Sakai, Kristen M. Raymond, Shannon K. McWilliams, Melissa J. Roark, Marie T. Banich

**Affiliations:** 1 Division of Substance Dependence, Psychiatry Department, University of Colorado Denver, Denver, Colorado, United States of America; 2 Institute of Cognitive Science, Departments of Psychology and Neuroscience, University of Colorado Boulder, Boulder, Colorado, United States of America; George Mason University, UNITED STATES

## Abstract

**Background:**

Accidental injury and homicide, relatively common among adolescents, often follow risky behaviors; those are done more by boys and by adolescents with greater behavioral disinhibition (BD).

**Hypothesis:**

Neural processing during adolescents' risky decision-making will differ in youths with greater BD severity, and in males vs. females, both before cautious behaviors and before risky behaviors.

**Methodology/Principal Findings:**

81 adolescents (Patients with substance and conduct problems, and comparison youths (Comparisons)), assessed in a 2 x 2 design (Patients:Comparisons x Male:Female) repeatedly decided between doing a cautious behavior that earned 1 cent, or a risky one that either won 5 or lost 10 cents. Odds of winning after risky responses gradually decreased. Functional magnetic resonance imaging captured brain activity during 4-sec deliberation periods preceding responses. Most neural activation appeared in known decision-making structures. Patients, who had more severe BD scores and clinical problems than Comparisons, also had extensive neural hypoactivity. Comparisons' greater activation before cautious responses included frontal pole, medial prefrontal cortex, striatum, and other regions; and before risky responses, insula, temporal, and parietal regions. Males made more risky and fewer cautious responses than females, but before cautious responses males activated numerous regions more than females. Before risky behaviors female-greater activation was more posterior, and male-greater more anterior.

**Conclusions/Significance:**

Neural processing differences during risky-cautious decision-making may underlie group differences in adolescents' substance-related and antisocial risk-taking. Patients reported harmful real-life decisions and showed extensive neural hypoactivity during risky-or-cautious decision-making. Males made more risky responses than females; apparently biased toward risky decisions, males (compared with females) utilized many more neural resources to make and maintain cautious decisions, indicating an important risk-related brain sexual dimorphism. The results suggest new possibilities for prevention and management of excessive, dangerous adolescent risk-taking.

## Introduction

A "propensity for risk-taking" [1] rises dramatically across adolescence, facilitating key adolescent tasks: increasing independence from parents, bonding to and competing with age mates, initiating sexual roles, and preparing for employment and parenthood. However, risky behaviors, defined as behaviors that may unpredictably result in either rewards or punishments, also include high-dose drug use, drunk driving, and carrying weapons; such behaviors commonly precede accidental injuries and homicides, the two leading causes of death in American adolescents [2].

Despite their dangers, risky behaviors usually have cautious alternatives, requiring risky-or-cautious decision-making. In situations permitting either (but not both) a risky or a cautious behavior, "risky-or-cautious decision-making" is whatever neural activity leads an adolescent to choose to do one alternative rather than the other (it also could mean whatever thinking leads to the choice, but thoughts are less relevant in this study). The definition is agnostic regarding the nature of that neural activity, but this report aims to clarify that nature and to determine whether it differs in different groups of adolescents.

### Age and Sex

A "dual systems theory" suggests that adolescents' increasing risk-taking reflects differing age-related development of brain systems. Rapidly developing reward structures, and slower-developing inhibitory structures, apparently bias adolescents' decisions toward riskiness [3,4].

The sexes differ in risk-taking. More boys than girls drive after drinking alcohol, "rarely or never" wear seatbelts, carry guns or other weapons, and die from accidents or homicides [5]. Boys commit three-fourths of America's juvenile violent crimes [6]. Similarly, adult males have a higher prevalence of antisocial and substance use disorders, reflecting higher mean levels of a risk-taking "externalizing" trait [7].

Conservation across many mammalian species of more risky, aggressive behavior in males [8] suggests a biological origin, and sexual dimorphism of brain and behavior are clearly related [9,10]. Human male and female brains do differ considerably [11,12], and brain imaging may capture risk-related sex differences. For example, in a risk-taking task adult females had greater activity in insula and orbitofrontal cortex [13], while in a stop-signal task men had more activation in numerous areas [14]. However, despite adolescents' sex differences in risk-related mortality, risky behaviors, and brain structure, we are unaware of studies examining sex differences in their neural processing of risky decisions.

### Individual Influences

Different individuals vary from showing marked constraint to severe behavioral disinhibition (BD), which is "a highly heritable general propensity to not constrain behavior in socially acceptable ways, to break social norms and rules, and to *take dangerous risks*, *pursuing rewards excessively despite dangers of adverse consequences*" (italics added) [15,16]. Other names have been applied to this trait [17–19]; it appears in prepubertal children as excessive disinhibited and aggressive behavior that often predicts adult substance and criminal problems [20]. Impulsivity, sensation-seeking, and some signs of attention-deficit/hyperactivity disorder (ADHD) may develop, frequently preceding the early-adolescent onset of the substance and conduct problems characterizing adolescent BD [16].

Such adolescents take excessive risks in real life (early sexual behavior, substance use, fighting, using weapons, thievery) and in laboratory tasks [21,22]. Behavioral disinhibition's mix of conduct and substance problems, ADHD symptoms, and novelty-seeking has an estimated heritability of 60–80 percent [23–25]; “what parents pass on to their … offspring is a non-specific, genetic liability to multiple externalizing disorders" [26].

### Role of the Brain

The brain apparently mediates that genetic liability. Youths with serious conduct problems reportedly have gray-matter volume deficiencies in several decision-related regions [27–32] (some disagree [33]). Blair [34] even proposes two types of conduct disorder, based in part on differing amygdala function.

Also, childhood brain P300 abnormalities [16], as well as Go/No-Go hypoactivity in several brain regions [35], both predict adolescent conduct, alcohol, and other drug problems. Moreover, during reward anticipation, risk-taking adolescents have less reward-system activation than others [36]. Low or otherwise atypical neural activity also is found in youngsters with BD-related conduct disorder, ADHD, or oppositional defiant disorder [37–41]. Even children just having adult relatives with alcohol use disorder show regional hypoactivity during decision-making [42].

Some of those studies, however, did not use risky-decision tasks, some obscured possible sex differences by mixing male and female participants, and some selected "pure" cases (*e*.*g*., conduct disorder without substance use disorders). Such selection, despite certain advantages, potentially misses youths with more severe behavioral disinhibition, since by definition just one of these disorders comprises less severe BD than several occurring together.

We previously [43] examined neural processing of risky-or-cautious decision-making *only in boys* who had BD-related conduct and substance problems; many additionally had ADHD symptoms. Participants made repeated decisions between doing a cautious behavior that always earned a little money, or a risky behavior that would either win or lose more money; the probability of winning started high but declined steadily. In analyses averaging across both risky and cautious decisions these boys showed significant hypoactivation in numerous brain regions. However, analyzing the processing preceding risky responses separately from that preceding cautious responses might better have clarified decision processing, and our recent unpublished analyses do suggest sufficient power to examine these response types separately. Moreover, sex differences clearly warrant investigation.

Accordingly, we now present new analyses of the neural processing preceding risky behaviors, and separately, of that preceding cautious behaviors, in 81 adolescents. They include female patients with serious conduct and substance problems and comparison females, together with the analogous male groups that had undergone different analyses in Crowley et al [43]. We hypothesized that neural processing during adolescent decision-making would differ in youths with high vs. low BD severity, and in males vs. females, both before cautious behaviors, and separately, before risky behaviors.

We had no data to predict which areas would activate more before risky, and which before cautious, but we predicted that areas activating in analyses combining those choices [43] would be involved; orbitofrontal and dorsolateral prefrontal cortices, anterior cingulate, basal ganglia, insula, amygdala, hippocampus, and cerebellum had activated less in patients in those combined analyses.

## Methods

### Ethics Statement

The Colorado Multiple Institutional Review Board approved all procedures. After explanation of procedures 18 year-old participants provided informed written consent; those <18 years old provided informed written assent and parents provided informed written consent. Participants self-identified their racial/ethnic categories from a government list.

### Participants, Assessments

The 81 participants were males and females, ages 14–18 years (inclusive) with IQ ≥ 80, without known MRI contraindications, history of lengthy unconsciousness, neurological illness, or neurosurgery. They received $50 payment, plus earning $6.35 (mean) in the behavioral task.

Patients’ inclusion criteria were: in treatment in our program (usually after juvenile justice or social service referral); antisocial problems including some DSM-IV [44] conduct disorder symptoms; DSM-IV non-tobacco substance use disorder; and urine and saliva tests drug-free ≥7 days before assessment. Patients’ exclusion criteria were: psychosis; current risk of suicide, violence, or fire setting; or positive urine pregnancy test. To reduce confounds from prolonged treatment, we also excluded patients in treatment and abstinent ≥ 30 days. Of patients, 28 males and 54 females enrolled; 20 males and 21 females completed all procedures. We enrolled more female patients because, while most boys were in stable residential treatment, all girls received less-constraining outpatient treatment and many relapsed or left before scanning. Nevertheless, the remaining male and female patients had remarkably similar severity of disorders (see Results).

Most comparison youths were recruited after phone contacts by a telemarketing company, but 9 volunteered after word-of-mouth or online advertising. Comparison youths' inclusion criteria for age and IQ were like patients’, and all lived in zip code areas that frequently contribute patients to our program. Exclusion criteria were: psychosis; serious court convictions or substance-related problems; physical illness; substance-positive biological tests; DSM-IV conduct disorder (past year); non-tobacco substance dependence; or positive urine pregnancy test. As samples accumulated, we adjusted comparison youths' recruitment (*e*.*g*., seeking older females) to maintain patient-comparison similarity. Twenty-five males and 26 females enrolled; 20 of each completed all procedures.

Assessments were done between April 2007 and November 2011. Males' recruitment was completed 42 months before females'.

Psychosocial assessments, done several days before fMRI's, typically required 2 hours for comparison youths and 3 for patients (who reported more symptoms). Assessments (references in [43]) were: Child Behavior Checklist (CBCL) and Youth Self Report (YSR) for symptom severity of ADHD, anxiety, and depression; Diagnostic Interview Schedule for Children (DISC- IV) for conduct disorder symptoms and diagnoses; Composite International Diagnostic Interview-Substance Abuse Module (CIDI-SAM) for DSM-IV abuse or dependence for 11 substance categories; Peak Aggression Rating Scale for aggression severity; Carroll Self-Rating Scale for depression severity; Synergy Interview for education, legal issues, and medical/ psychological history; Modified Hollingshead-Redlich Social Class Rating; Wechsler Abbreviated Scale of Intelligence (WASI) Vocabulary and Matrix Reasoning for IQ estimates; Eysenck Junior Impulsiveness Scale; and handedness preference. Before scanning patients underwent frequent clinical urine and saliva tests for drugs; comparison youths had them about one week, and again immediately, before scanning.

### Analytic Plan

After adjusting for age and IQ we examined neural activation in the 4-sec deliberation period preceding responses, separately for risky and cautious responses, with 2 x 2 analyses of variance (ANOVA), seeking main effects of gender and group, and gender x group interactions. Variables were evaluated for outliers and approximately normal distributions. ANOVAs, Fisher Exact tests, and Pearson and Kruskall-Wallis Chi-square tests compared groups on demographic and clinical characteristics, in-task behaviors, and responses to debriefing Visual Analogue Scales. Differences between pairs of groups were only evaluated if the sex by group interaction (or overall group test for non-continuous variables) was significant. For the estimated lines graphing the groups' risky right presses across three 30-trial runs a mixed model with random intercepts and slopes evaluated effects of group, sex, and their interaction. All statistical tests were two-tailed.

### Estimating Abstinence Duration

Among patients 12 boys and 8 girls, most referred from strictly controlled environments, denied substance use in the 30 days before admission and produced substance-free urine samples from admission until scanning. For them we estimated abstinence duration as: (30 days) + (number of days between admission and imaging). Others’ abstinence duration was the number of days with in-treatment negative urine samples before imaging.

One male and one female comparison participants reported regular (≥ monthly) tobacco smoking. Among patients 20 of 21 females and all males reported regular smoking before admission, but 14 of those boys were now in residential treatment that vigorously suppressed smoking. Thus, we conservatively estimated that 6 male and 20 female patients, and one male and one female comparison youths, had smoked within a few days before imaging. No participants smoked during the hour before scanning.

### Severity of Behavioral Disinhibition

Participants' BD scores were computed from a composite of 4 measures of disinhibited behavior: lifetime conduct disorder symptom count; lifetime substance abuse and dependence symptom counts, summed across CIDI-SAM's named drug classes to create a combined substance use disorder score; and parent-provided CBCL problem scores for Inattention and (separately) Hyperactivity/Impulsivity. Four patients' parents (one boy, three girls) did not provide CBCL data; we substituted comparable YSR information.

To standardize BD scores against community adolescents we randomly drew from a previously-recruited community sample of Colorado adolescent twins and their siblings [45] one youth per family (208 females, 187 males). Their mean age 16.8 (±0.72) years placed them approximately in the age range of our fMRI participants. We estimated those youths' BD severity from the variance shared among the above four behavioral variables, using a confirmatory factor analysis within that sample to generate factor loadings for each variable. Resulting composite scores were expressed as community-sample *z*-scores. Using those factor-weights from the community sample, each fMRI participant's composite BD score was computed as the sum of the four factor-weighted behavioral scores, and those BD scores were expressed as *z*-score deviations from the community sample.

### Decision-Making Behavioral Task

**Fig 1:**
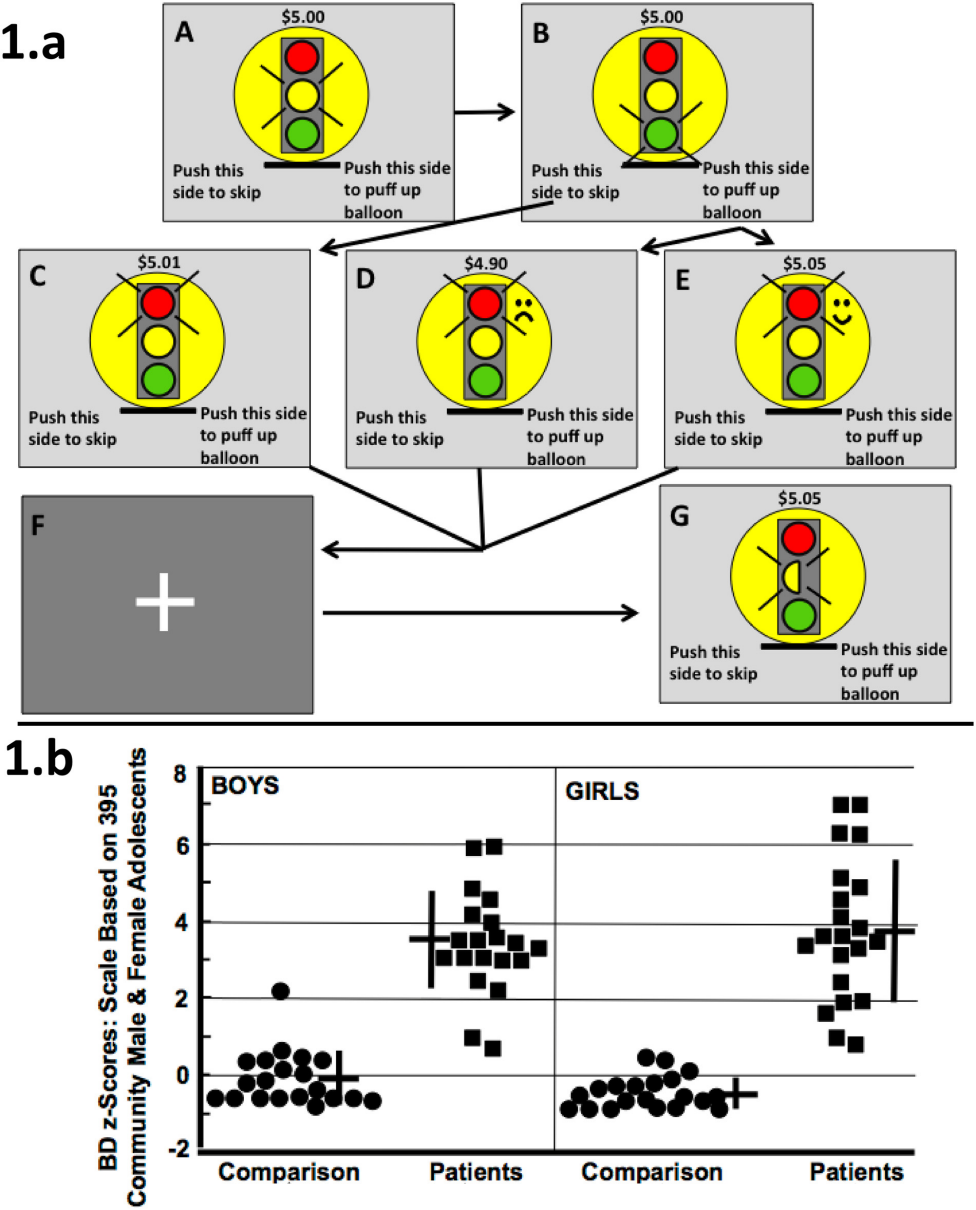
Game; Participants' Behavioral Disinhibition Scores. **(a) Colorado Balloon Game**. *A*: Decision Trial begins, yellow illuminated 4 sec. *B*: Chosen press executed during 0.5-second green light. Next, red light signals outcome, 3.5 sec. If left press in *B*, then *C* (counter increases 1 cent, dull sound, no change in balloon). If right press in *B*, then either *D* (counter decreases 10 cents, frowney face, popping sound, balloon shrinks), or *E* (counter increases 5 cents, smiley face, coin-drop sound, balloon puffs up). *F*: Fixation screen 2–4 sec. *G*: Directed Trial begins with 4-sec yellow half-light. Illuminated side (left here) indicates side to press when light turns green. Counter: current earnings. Then, the B-F sequence from the paired Decision Trial follows exactly, but participant knows that "the computer is playing the game now", and that only the counter increase of 2 cents upon the directed press affects participant. **(b) 81 Participants’ BD Scores**. Crosses: means, standard deviations.

After mock-scanner training, during rapid event-related fMRI participants played the Colorado Balloon Game [43], conceptually based on Newman's game [46] and little-related (despite others' suggestions [47]) to Lejuez's Balloon Analogue Risk Task [21]. Participants began with $5 and kept any earnings. In 90 “Decision Trials” they considered during a 4 sec yellow light whether next to do a cautious or a risky behavior (Fig 1a.A). Then, during a 0.5 sec green light (Fig 1a.B) they made the chosen response: a left finger-press (cautious) always earned 1 cent; a right press (risky) either won 5 or lost 10 cents. Participants were not informed that, across the game, probabilities for right-press "wins" gradually fell from 0.78 to 0.22. Next, a 3.5 sec red light signaled outcomes (including new dollar totals) from a cautious left response (Fig 1a.C), or a risky right-response loss (Fig 1a.D) or win (Fig 1a.E). Finally, a 2–4 sec. “jittered” fixation screen appeared (Fig 1a.F).

A “Directed Trial” (Fig 1a.G) followed each Decision Trial. Each Directed Trial shared identical timing and visual and auditory stimuli (Fig 1a.A–1a.F) with its paired, preceding Decision Trial. Directed Trials required no decisions; a yellow half-light signaled both onset of a Directed Trial and whether to press right or left (the same side chosen by the participant in the paired Decision Trial), and the youth's only task was to make the directed right or left press during the green light, thereby earning 2 cents. The 90 pairs of Decision and Directed Trials were divided into 3 runs, each with 30 identically-ordered pairs.

In Decision Trials the high initial probability of winning on risky responses aimed to make risky responses prepotent, as in [46], while small 1-cent rewards (with muted auditory and visual stimuli; Fig 1a.C) aimed to make cautious responses less attractive. Then, with the probability of reward from risky responses declining we reasoned that cautious responding would require neural inhibition of prepotent risky responses. Meanwhile, in Directed Trials the risk-free, entirely predictable 2-cent reward only aimed to assure responding by occasionally uncooperative patients.

We assessed brain activity during the 4-sec yellow-light pre-response deliberation period, separately analyzing deliberations ending in cautious or risky responses. For those 4-sec periods we subtracted Directed-Trial brain activation (Fig 1a.G) from Decision-Trial activation (Fig 1a.A) to minimize visual, auditory, and motor-related activation, while highlighting pre-response decision-making activation.

Post-session debriefings addressed in-magnet experiences and game strategies. On Visual Analogue Scales participants rated Decision, and separately Directed, Trials for computer-directed vs. self-choice of responses, and for happy-sad reactions to win or loss feedback.

### Image Acquisition and Analysis

In a 3T GE MRI scanner participants had a 3D T1 anatomical scan (IR-SPGR, TR = 9 ms, TE = 1.9 ms, TI = 500 ms flip angle = 10°, matrix = 256×256, FOV = 220 mm^2^, 124 1.7 mm thick coronal slices; 9 min 12 sec), followed by 3 echo-planar (EPI) runs (TR = 2000ms, TE = 26 ms, flip angle = 70°, FOV = 220 mm^2^, 64^2^ matrix, 36 slices, 4 mm thick, no gap, angled parallel to the planum sphenoidale, voxel size = 3.43 x 3.43 x 4 mm^3^), separated by 1-minute rests. Additionally, we acquired one IR-EPI volume to improve co-registration. Fast z-shimmed acquisition reduced inferior frontal susceptibility artifact [48], showing robust orbitofrontal activation.

We conducted realignment, coregistration, spatial normalization, and smoothing (with 6mm full-width half-maximum Gaussian kernel). For within-subject fMRI analyses we fitted preprocessed data with the general linear model (GLM) of Statistical Parametric Mapping (SPM) software, filtering low frequency noise, correcting for temporal autocorrelation using the autoregressive model (AR(1)), convolving with a single canonical HRF signal. A 128-s high pass filter removed signal drift and low-frequency fluctuation. The GLM model included separate trial periods, Decision (risky-or-cautious) and Outcome (win or loss, not reported here). We generated single-subject contrast maps with SPM8, analyzing brain-function differences in contrasts of interest (*e*.*g*., Risky_*Decision Trial*_ or Risky_*Directed Trial*_) as fixed effects.

Between-subject whole-brain analyses compared groups' single-subject SPM8 contrast maps, using SPM8's random effects models. We used SPM8's 2x2 (ANOVA) model with contrast maps from the following 4 groups: Male Patients, Female Patients, Male Comparisons, and Female Comparisons, with age and IQ added as nuisance covariates (without any centering or interaction).

### Selecting and Interpreting Multiple-Comparisons Corrections

To compare groups' (Decision Trial – Directed Trial) activations, we sought significant differences with whole-brain analyses, controlling for multiple comparisons with cluster-level family-wise error correction (CL-FWE). Using 6 mm full-width-half-maximum smoothing, 1000 Monte Carlo simulations estimated CL-FWE whole-brain significance levels; clusters with >96 voxels, each activated at *p*_uncorr_<0.005, provided whole-brain *p*_corr_<0.05 [43,49].

To test main effects and interactions we first produced statistical maps at the threshold of *p*_uncorr_<0.005 at voxel level with an extent threshold of >96 contiguous voxels per cluster, and then tested for Group x Sex interactions. We next examined main effects of group and gender with the same threshold of *p*_uncorr_<0.005, >96 contiguous voxels, after excluding regions of interaction found in Step 1. We present tables of all main effects after excluding interactions (*i*.*e*., Comparison participants > patients, patients > comparisons, males > females, and females > males). These analyses, using the contrast [(Decision Trial) – (Directed Trial)]_*GroupA*_ – [(Decision Trial) – (Directed Trial)]_*GroupB*_, asked whether the difference in activation intensities between comparison youths vs. patients, or males vs. females significantly differed from zero.

### Four Possible Confounds

We examined potential confounds with ‘‘glass-brain” images that show all beyond-threshold areas of activation as "shadows" cast on a 2-dimensional surface, simplifying pattern recognition in large data sets. We present glass brain images addressing the two main effects studied (comparison vs. patient participants; males vs. females). For example, for the comparison vs. patient main effect the glass brains show five sets of activation data: *(a)* all comparison vs. all patient participants after statistically adjusting activation intensity for age and IQ; *(b)* like (a), but also statistically adjusting for depression severity (Carroll Rating Scale); *(c)* like (a), but excluding left-handed participants (2 patient males, 1 comparison male, 3 patient females, no comparison females); *(d)* like (a), but excluding medicated participants (Table 1); and *(e)* like (a), but excluding recent regular tobacco smokers.

**Table 1:** Individual Participants' Medications.

Group	Females	Males
Patient	Bupropion, ziprasidone	Amphetamine + dextroamphetamine, risperidone
Patient	Citalopram, lamotrigine	Fexofenadine
Patient	Oxcarbazepine, levonorgestrel + ethinyl estradiol	Fluoxetine, quetiapine
Patient	Bupropion	Unidentified ‘‘ulcer drug"
Patient	Fluoxetine	Methylphenidate
Patient	Aripiprazole	Unidentified "asthma inhaler"
Patient	Unidentified common-cold remedy	
Patient	Aripiprazole	
Comparison	Fluoxetine	Topiramate
Comparison	Lansoprazole, Spironolactone	Albuterol inhaler
Comparison	Ibuprofen	Amphetamine + dextroamphetamine
Comparison	Unidentified "birth control"	Amphetamine + dextroamphetamine
Comparison	Unidentified "birth control"	

Footnote:

^+^ indicates a combination medication

### Anatomic Considerations

We refer to certain ‘‘broad regions” [43]. Dorsolateral prefrontal cortex (PFC) includes parts of Brodmann Areas (BA) 46, 9, 8, and 6 [50]. Portions of BA 6 on the medial cerebral wall are "pre-supplementary motor area" if rostral to, or "supplementary motor area" if caudal to, the anterior commissure [51]. Subcallosal gyrus includes BA 25 and parts of BA 24 and 32 [52]. Our tables list structures (from the online Münster atlas [53] contributing ≥10 voxels to a cluster. Rarely, clusters appeared outside gray matter, perhaps reflecting registration errors; clusters <3mm from the nearest gray matter are labeled there, while those ≥3mm away are not reported.

## Results

### Participants

We standardized the combined-sex BD scores of the 187 boys and 208 girls in our larger community sample (mean 0; standard deviation 1); then, the community sample's male mean = + 0.13 ± 1.07; female mean = - 0.16 ± 0.89 (*t* = 3.98; *p* < 0.001). Applying that scale in our fMRI sample revealed large patient-comparison group differences in BD scores (Fig 1.b; Table 2), and those differences were reflected in nearly every other clinical measure (Table 2).

**Table 2:** Demographic and Clinical Information.

**Measures Assessed by ANOVA**	**20 Cmp**	**20 Cmp**	**21 Patient**	**20 Patient**	**Pt:Cmp Group Difference**		**Male:Female Sex Difference**		**Group x Sex Interaction**	
	**Females**	**Males**	**Females**	**Males**	**Statistic** ^1^ ^,^ ^2^	***p*** ^3^	**Statistic** ^1^	***p*** ^3^	**Statistic** ^1^ ^,^ ^2^	***p*** ^3^
Mean Age (SD)	16.7 (1.1)	16.5 (1.6)	16.2 (1.1)	16.5 (1.0)		NS		NS		NS
Behavioral Disinhibition Score: Mean (SD)	-0.49 (0.40)	-0.08 (0.69)	3.9 (2.0)	3.4 (1.3)	F(1,42.9) = 199.3	0.0005		NS		NS
Socio-economic Status Score: Mean (SD)^4^	34.9 (15.7)	34.5 (15.5)	46.6 (13.3)^5^	47.2 (18.3)	F(1,73) = 11.4	0.001		NS		NS
Eysenck Impulsiveness Score: Mean (SD)	5.0 (3.7)^w^	6.7 (4.5)^x^	15.9 (5.0)^w^ ^,^ ^y^	11.9 (6.0)^x^ ^,^ ^y^		NA		NA	F(1,77) = 6.94	0.010
Youth Self Report: Conduct Problems Score: Mean (SD)	2.2 (2.0)	3.2 (2.4)	9.9 (4.9)	10.8 (4.2)	F(1,55.9) = 93.2	0.0005		NS		NS
Conduct Disorder Lifetime Symptom count: Mean (SD)	0.35 (0.59)	0.45 (0.60)	5.4 (3.3)	6.8 (2.3)	F(1,39.2) = 160.7	0.0005		NS		NS
CBCL^6^ Attention Problems Scale score: Mean (SD)	1.4 (1.9)^w^ ^,^ ^x^	4.1 (3.6)^w^	7.8 (4.6)^x^	6.6 (4.6)		NA		NA	F(1,63.0) = 5.30	0.025
CBCL^6^ Anxiety-Depression Scale score: Mean (SD)	1.7 (1.4)	1.9 (3.7)	7.0 (3.5)	4.7 (4.6)	F(1,57.8) = 27.7	0.0005		NS		NS
IQ Full-Scale score estimate: Mean (SD)	103.5 (10.4)	104.9 (9.0)	95.4 (9.9)	97.3 (8.9)	F(1,77) = 13.5	0.0005		NS		NS
**Measures Assessed by Other Procedures**	**20 Cmp**	**20 Cmp**	**21 Patient**	**20 Patient**	**Overall Difference**					
	**Females**	**Males**	**Females**	**Males**	**Statistic** ^1^ ^,^ ^2^	***p*** ^3^				
Ethnicity: Non-Hispanic Euro-American (n)	13	15	14	12	**X** Sq	NS				
Ethnicity: Other racial-ethnic groups (n)	7	5	7	8	**X** Sq	NS				
Aggression Score^7^	0 (0)^w^ ^,^ ^x^	0.0 (0–4)^w^ ^,^ ^y^	6.0 (0–9)^x^	7.0 (0–9)^y^	K-W **X** Sq (3) = 53.6	0.0005				
Conduct Disorder Lifetime Diagnosis (n)	1^w^	1^x^	16^w^	19^x^	x Sq (3) = 54.2	0.0005				
Carroll Depression Rating Score^7^	3.0 (0–11)^w^	3.0 (0–15)^x^	9.0 (3–22)^w^	7.0 (0–28)^x^	K-W **X** Sq (3) = 24.2	0.0005				
Substance Dep Symptoms, Across Drug^7^	0.0 (0–5)^w^	0.0 (0–3)^x^	11.0 (0–30)^w^	11.0 (1–31)^x^	K-W **X** Sq (3) = 63.4	0.0005				
Recent Regular Smokers (n)^8^	1^w^	1	20^w^ ^,^ ^x^	6^x^	**X** Sq (3) = 49.8	0.0005				
Tobacco Dependence (n)	1^w^	1^x^	11^w^	13^x^	**X** Sq (3) = 27.4	0.0005				
Alcohol Abuse (n)	0	0^w^	4	8^w^	FE = 16.0	0.0005				
Alcohol Dependence (n)	0^w^	0^x^	14^w^	8^x^	**X** Sq (3) = 33.1	0.0005				
Cannabis Abuse (n)	0^w^	0^x^	7^w^	7^x^	FE = 17.1	0.0005				
Cannabis Dependence (n)	0^w^	0^x^	12^w^	10^x^	**X** Sq (3) = 29.7	0.0005				
Participants with Other Substance Use Disorders (n)^9^	0^w^	0^x^	11^w^	9^x^	FE = 28.3	0.0005				
Lifetime Court Appearances^7^	0 (0)^w^	0 (0)^x^	5.0 (0–28)^w^	9.0 (1–50)^x^	K-W **X** Sq (3) = 60.2	0.0005				
Lifetime Admissions to Detention or Jail^7^	0 (0)^w^	0 (0)^x^	1.0 (0–6)^w^	2.0 (0–20)^x^	K-W **X** Sq (3) = 53.0	0.0005				
Days on Probation, Last 6 Months^7^	0 (0)^w^	0 (0)^x^	0.0 (0–180)^w^ ^,^ ^y^	180 (0–180)^x^ ^,^ ^y^	K-W **X** Sq (3) = 45.1	0.0005				

Abbreviations:

ANOVA, analysis of variance. CBCL, Child Behavior Checklist. Cmp, Comparison. Dep, dependence. FE, Fisher exact test. K-W **X** Sq, Kruskall-Wallis Chi-square. n, number. NA, not assessed. NS, not significant. Pt, patient. SD, standard deviation. SES, socioeconomic status. **X** Sq, Pearson Chi-square.

Footnotes:

^1^Not provided if test is NS.

^2^If interactions/overall test was significant, 4 post-hoc comparisons, as shown by arrows: Cmp Male ←→ Pt Male Cmp Female ←→ Pt Female

^3^NS: p > 0.05, two-tailed. NA: Not assessed because of significant interaction.

^4^Highest class = V. Comparison mean score falls in Class IV, patient in Class III.

^5^n = 18.

^6^In one patient with no CBCL, YSR score was substituted for CBCL.

^7^Median (range) provided for very skewed values.

^8^"Regular" is at least monthly. All patient males had been regular smokers, but 14 were now in a smoke-free residential treatment facility.

^9^DSM-IV abuse or dependence on any drug not listed above.

^w-y^Within one row values sharing a superscript are significantly different.

### Abstinence Duration

We estimated patients' pre-scanning abstinence duration for non-tobacco drugs as: girls, mean 35.6 (range 7–63) days; boys, 38.6 (range 9–59) days. In the 30 days before imaging two comparison youths used cannabis on one day; two used alcohol on one day and one did on two days; none reported use in the week before scanning, and biological tests just before scanning revealed no alcohol or non-prescribed drugs.

### Participants' Understanding of the Game

Inspection of the Self-rated Visual Analogue Scales (Table 3) suggests that in Decision Trials all groups felt happiness when balloons puffed up (actual wins) and sadness when they popped (actual losses), whereas the Directed Trials' sham wins and losses produced more neutral feelings; crucially, there was no significant Patient:Comparison or Male:Female difference in these emotional responses (Table 3). Also, the four groups similarly understood that they themselves decided which button to press in Decision Trials, and that the computer told them which to press in Directed Trials (Table 3). Thus, participants understood the game, their understanding did not vary among the groups, and actual and sham wins and losses produced the intended emotional responses.

**Table 3:** Participants' Behavior in Task.

**Measures Assessed by ANOVA**	**20 Cmp** ^1^	**20 Cmp** ^1^	**21 Patient** ^1^	**20 Patient** ^1^	**Test**	**Pt:Cmp Difference**		**Male:Female Difference**		**Group x Sex Interaction**	
	**Females**	**Males**	**Females**	**Males**		**Statistic** ^6^	***p***	**Statistic** ^6^	***p***	**Statistic** ^6^	***p***
Happy, Balloon Puffs Up, Decision Trial^2^	14.7 (11.8)	14.4 (16.8)	17.1 (16.4)	24.0 (18.2)	ANOVA		NS		NS		NS
Happy, Balloon Pops, Decision Trial^2^	75.6 (16.4)	69.1 (25.8)	68.2 (19.3)	61.7 (16.2)	ANOVA		NS		NS		NS
Happy, Balloon Puffs Up, Directed Trial^2^	37.9 (15.8)	40.3 (17.6)	34.6 (20.6)	34.8 (18.7)	ANOVA		NS		NS		NS
Happy, Balloon Pops, Directed Trial^2^	49.0 (12.2)	45.7 (13.9)	39.8 (21.0)	42.8 (21.1)	ANOVA		NS		NS		NS
Total Risky Responses	46.5 (9.9)	52.4 (8.7)	41.4 (15.0)	52.2 (10.3)	ANOVA		NS	F(1,77) = 11.1	0.001		NS
Total Cautious Responses	41.5 (10.0)	36.2 (9.1)	44.5 (15.1)	35.4 (9.4)	ANOVA		NS	F(1,77) = 8.43	0.005		NS
Risky Wins	25.9 (5.4)	29.9 (4.6)	23.3 (8.0)	28.3 (5.2)	ANOVA		NS	F(1,77) = 11.4	0.001		NS
Risky Losses	20.6 (5.1)	22.5 (4.8)	18.1 (7.6)	23.8 (6.0)	ANOVA		NS	F(1,69.4) = 8.40	0.005		NS
Total $ Earnings in Game	6.40 (0.43)	6.37 (0.43)	6.51 (0.61)	6.13 (0.53)	ANOVA		NS		NS		NS
Decision Trial, Risky Response Time^3^	292.5(28.2)^w^	252.5 (27.3)^w^	276.6 (24.9)	263.9 (27.4)	ANOVA		NA		NA	F(1,77) = 5.23	0.025
Decision Trial, Cautious Response Time^3^	279.7(26.1)	254.7 (29.9)	287.2 (30.4)	258.7 (31.1)	ANOVA		NS	F(1,77) = 16.7	0.001		NS
**Measures Assessed by K-W Chi Square**	**20 Cmp**	**20 Cmp**	**21 Patient**	**20 Patient**	**Test**	**Overall Difference**					
	**Females**	**Males**	**Females**	**Males**		**Statistic** ^6^	***p***				
Who told me which to press, Decision Trial^4^ ^,^ ^5^	3.0 (0–54.5)	1.0 (0–64)	2.0 (0–29.4)	2.0 (0–65)	K-W	**X** ^2^	NS				
Who told me which to press, Directed Trial^4^ ^,^ ^5^	97.5 (35–100)	99.0 (1–100)	94.6 (1–100)	98.3 (56–100)	K-W	**X** ^2^	NS				
Total Missed Responses^5^	3.5 (0–15)^w^ ^,^ ^x^	1.0 (0–5)^w^ ^,^ ^y^	7.0 (2–30)^x^ ^,^ ^z^	2.0 (0–6)^y^ ^,^ ^z^	K-W	**X** ^2^(3) = 53.6	0.0005				

Abbreviations: As in Table 2.

Footnotes:

^1^Mean (Standard Deviation).

^2^"Really, really happy" = 0 mm; "Really, really sad" = 100 mm.

^3^Based on completed responses.

^4^"I told myself" = 0 mm; "the computer told me" = 100 mm.

^5^Median (Range) provided for very skewed values.

^6^If interactions/overall test was significant, 4 post-hoc comparisons, as shown by arrows: Cmp Male ←→ Pt Male Cmp Female ←→ Pt Female

^w-z^Within one row values sharing a superscript are significantly different.

### Behavior in the Game

**Fig 2:**
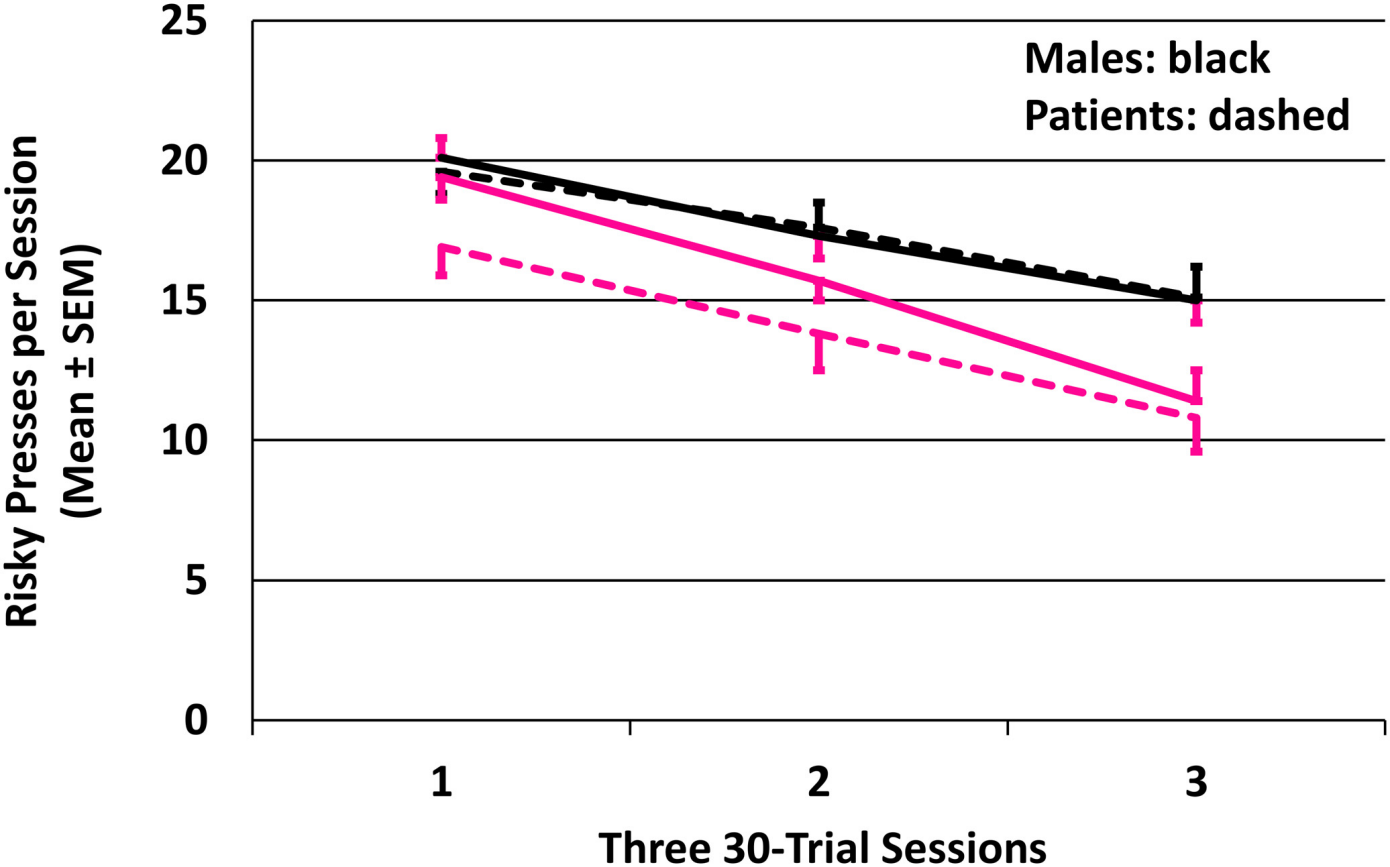
Decline in risky presses as probability of loss increased. Four groups: patient males, comparison males, patient females, comparison females. Raw (not fitted) data shown. Comparison males' error bars: red to reduce confusing overlaps. Trials required a choice: a cautious response earned 1 cent; a risky response either won 5 or lost 10 cents. Probability of winning after risky choices declined from 0.78 to 0.22 as game progressed.

Reflecting the steadily increasing chance of punishment for risky responses, across the session all 4 groups very significantly reduced their risky pressing (Fig 2; *p*'s < 0.0001; mixed model analysis jointly considering the four lines' elevations and slopes). The joint tests showed that, overall, females made fewer risky presses than males (F (2,77) = 7.14; *p* = 0.0014). Comparison and patient participants did not differ in risky pressing, nor was there a group x sex interaction.

So boys made more risky responses than girls. Comparison boys also were faster than comparison girls on risky responses, perhaps contributing to girls' significantly more frequent failures to respond in the required time (Table 3). A significant group x sex interaction for risky response time indicated that comparison boys were significantly faster than comparison girls, with patient boys and girls responding more similarly. Boys' more frequent (than girls') risky responses resulted in significantly more wins and losses (Table 3); total earnings, however, were not significantly different, perhaps because boys both won and lost more. Meanwhile, patient and comparison participants differed only in numbers of missed responses (Table 3).

### Processing Differences: Patient and Comparison Groups

**Fig 3:**
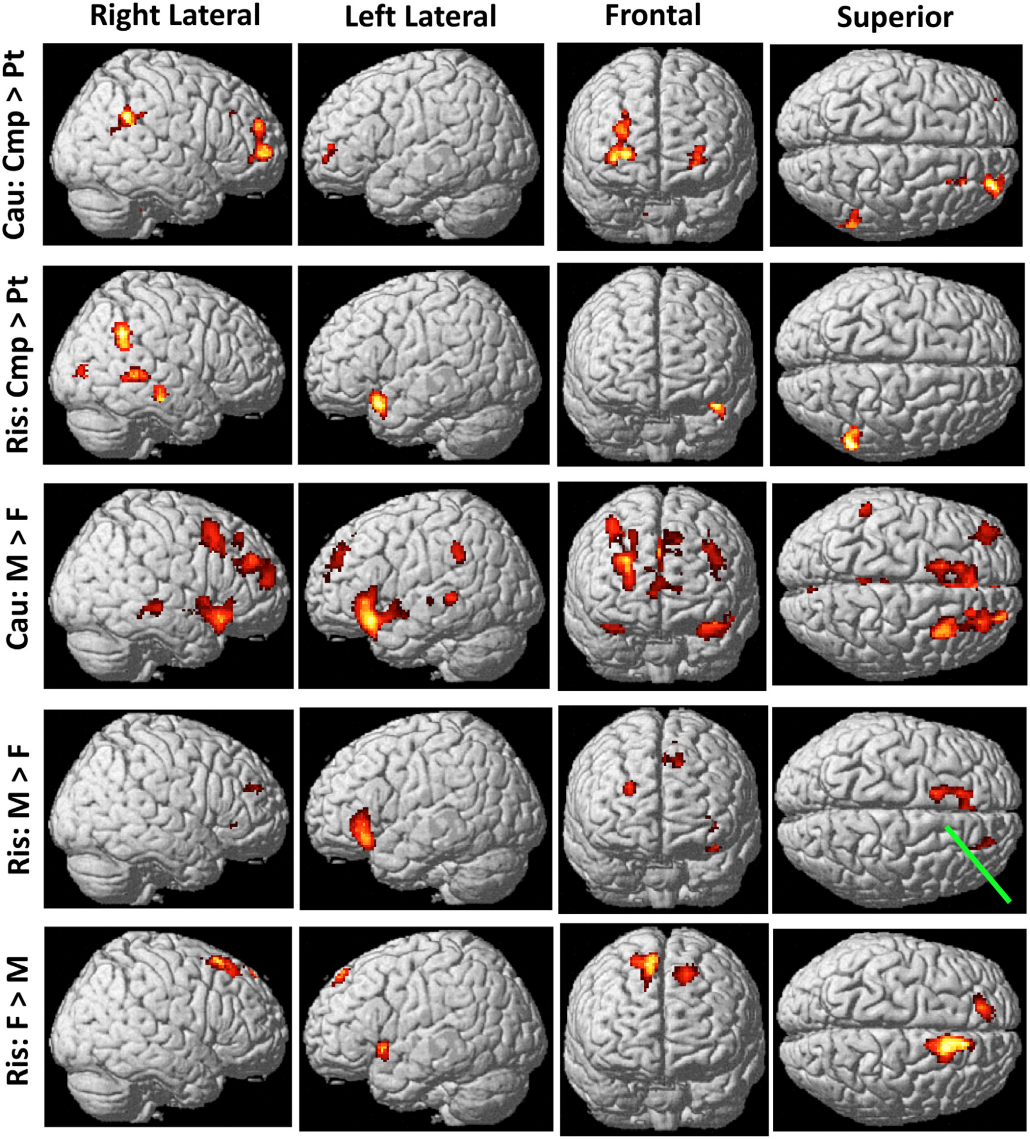
Cortical regions near scalp where groups' activations differed significantly. These regions are potentially accessible to transcranial direct current stimulation. Row labels: *Cau*, before cautious responses. *Ris*, before risky responses. *Cmp*, comparison participants. *F*, females. *M*, males. *Pt*, patients. Three rows of possible activations not shown: (a) Cau: Pt>Cmp and (b) Ris: Pt>Cmp, were both devoid of activation; (c) Cau: F>M had activation only at site marked by *Green pointer tip* in Ris: M>F. Significant t-values range from 2.64 (dark red) to 7.76 (white); details in Tables 4 and 5.

We examined neural activity differences among the four groups, using 2 sex X 2 group (patient, comparison) ANOVAs. Fig 3 shows all lateral, frontal, or superior cortical regions that had significant main effect differences.

Before cautious responses comparison participants' greater activity was mostly concentrated in one large, bilateral cluster including frontal pole, medial PFC, striatum, and thalamus (Table 4, All Cmp > All Pts). Before risky responses (Table 5, All Cmp > All Pts) comparison participants' greater activation was mostly temporal, parietal, and occipital, with fewer prefrontal regions involved. Patients' activation nowhere exceeded comparison youths' before either cautious or risky responses.

**Table 4:** The Groups' Activity Differences before Cautious Responses.

**All Males > All Females (Excluding Regions of Interaction)** ^a^								**All Females > All Males (Excluding Regions of Interaction)** ^a^							
**Structure**	**BA or**	**Cluster**	**x**	**y**	**z**	**t** ^d^	**Region or**	**Structure**	**BA or**	**Cluster**	**x**	**y**	**z**	**t** ^d^	**Region or**
	**Side** ^b^	**Size** ^c^					**Function**		**Side** ^b^	**Size** ^c^					**Function**
Sup Fr Gy	R8,10	1451	24	56	14	3.7	dlPFC, Fr Pole	Med Fr Gy	R8	99	12	18	52	3.7	mPFC
Mid Fr Gy	R6,8,9,10		26	18	40	5.2	dlPFC, Fr Pole	Sup Fr Gy	R8		10	18	54	3.4	mPFC
Med Fr Gy	R9		26	36	30	3.0	mPFC	Cing Gy	R32		12	20	46	2.7	mPFC
Med Fr Gy	R,L10,32	198	10	48	4	3.3	mPFC	**Total**		**99**					
Ant Cing Gy	R,L32		0	52	12	3.3	mPFC								
Sup Fr Gy	L10	193	-40	50	30	4.1	Fr Pole	**All Cmp > All Pts (Excluding Regions of Interaction)** ^a^							
Mid Fr Gy	L9,10		-40	44	30	3.0	dlPFC	**Structure**	**BA or**	**Cluster**	**x**	**y**	**z**	**t** ^d^	**Region or**
Med Fr Gy	R,L9. L8	1948	-12	40	24	4.2	mPFC		**Side** ^b^	**Size** ^c^					**Function**
Ant Cing Gy	R,L24,32		-12	28	28	6.6	mPFC	Sup Fr Gy	R,L10	3300	24	60	2	2.9	Fr Pole
Cing Gy	R,L32		-4	14	44	3.5	mPFC	Mid Fr Gy	R,L10		34	54	6	3.5	Fr Pole
Sup Fr Gy	L6,8		-12	12	56	3.4	mPFC, SMA	Med Fr Gy	R,L10,32		20	52	0	2.7	mPFC
Inf Fr Gy	R47	1057	34	20	-16	5.4	vlPFC	Ant Cing Gy	R,L25		-14	32	-4	2.8	mPFC
Sup Temp Gy	R22,38		44	12	-14	2.9	Temp	Claustrum	R		28	10	12	2.8	Sens Integ
Insula	R13		40	8	-2	3.7	Insula	Caudate	R,L		8	10	2	3	Striatum
Mid Fr Gy	L11,47	5238	-38	34	-4	4.1	OFC, vlPFC	Putamen	R,L		20	6	2	3.0	Striatum
Inf Fr Gy	L45,47		-36	28	-8	7.0	vlPFC	Glob Pall	R,L		18	-2	4	2.9	Striatum
Sup Temp Gy	L22,38		-44	12	-16	3.2	Temp	Thal	R,L		-14	-4	6	4.2	Thal
Subcall Gy	R25		12	10	-14	2.7	mPFC	Mid Fr Gy	R9	187	24	28	32	4.4	dlPFC
Ant Cing Gy	R,L25		6	10	-8	3.6	mPFC	Inf Par Lob	R40	229	50	-44	26	2.9	Par
Caudate	R		6	8	2	3.2	Striatum	Supramarg Gy	R40		52	-48	32	3.3	Par
Putamen	R,L		-20	6	-4	3.4	Striatum	Nodule	R,L	178	14	-44	-36	3.4	Cerebell
Hypothal	R,L		4	-2	-8	4.6	Limbic	Pons	R		14	-42	-40	2.8	Br Stem
Globus Pall	R,L		-18	-6	-6	3.1	Striatum	Uvula	R,L		-12	-62	-38	3.1	Cerebell
Subthal Nucl	R,L		10	-12	-4	3.1	Basal Ganglia	Pyramis	L		-16	-64	-38	2.8	Cerebell
Insula	L13		-40	-14	-4	3.1	Insula	**Total**		**3894**					
Thal	R,L		-6	-20	2	3.8	Thal								
Red Nucl	R,L		4	-20	-10	3.0	Br Stem	**Regions Showing Gender X Group Interactions**							
Parahip Gy	L28		-24	-24	-12	3.2	Limbic	**Structure**	**BA or**	**Cluster**	**x**	**y**	**z**	**t** ^d^	**Region or**
Claustrum	L		-32	-24	6	3.1	Sens Integ		**Side** ^b^	**Size** ^c^					**Function**
Hippoc	L		-30	-26	-10	2.7	Limbic	Precent Gy	R4	109	16	-32	58	3.8	Motor
Mid Br	R,L		-6	-28	-4	4.1	Mid Br	Postcent Gy	R3		26	-32	62	2.7	Motor
Mid Temp Gy	L21,22		-50	-38	-2	3.5	Temp	Paracent Lob	R6		12	-32	58	3.3	Motor, Sens
Cing Gy	L31	255	-10	-24	32	3.7	Limbic	**Total**		**109**					
Paracent Lob	L31		-2	-24	46	2.7	Motor, Sens								
Mid Temp Gy	R21,22	365	46	-26	-8	4.0	Temp								
Sup Temp Gy	R22		48	-26	-2	3.4	Temp								
Supramarg Gy	L40	152	-54	-42	36	3.9	Parietal								
Inf Par Lob	L40		-52	-42	40	3.3	Parietal								
Paracent Lob	L5	102	-6	-44	52	3.5	Motor, sens								
Precuneus	L7		-6	-48	48	2.7	Med Par								
Cing Gy	R31	186	18	-40	36	4.0	Limbic								
Precuneus	R7,31		16	-48	34	2.7	Med Par								
Lingual Gy	L19	116	-30	-58	-6	4.0	Occip								
Parahip Gy	L19		-26	-54	-10	2.7	Limbic								
Precuneus	L7,31	140	-16	-74	28	4.4.	Parietal								
Cuneus	L18		-18	-78	22	2.7	Occip								
Cuneus	R7,18,19	129	6	-82	26	3.2	Occip								
Precuneus	R7,31		10	-68	30	3.1	Med Par								
**Total**		**11530**													

Abbreviations (Tables 4 and 5):

Ant, anterior. Br, brain. Cerebell, cerebellum. Cing, cingulate. Cmp, comparison participants. dl, dorsolateral. Fr, frontal. Glob Pall, globus pallidus. Gy, gyrus. Hippoc, hippocampus. Hypothal, hypothalamus. Inf, inferior. L, left. Lob, lobule. m or Med, medial. Nucl, nucleus. Pt, patients. Occip, occipital. OFC, orbitofrontal cortex. Par, parietal. Paracent, paracentral. Parahip, parahippocampal. PFC, prefrontal cortex. Postcent, postcentral. Precent, precentral. R, right. Sens, sensory. Sens Integ, sensory integration. SMA, supplementary motor area. Subcall, subcallosal. Subthal, subthalamic. Sup, superior. Supramarg, supramarginal. Temp, temporal. Thal, thalamus. vl, ventrolateral.

Footnotes (Tables 4 and 5):

^a^Contrast: [(Decision Trial - Directed Trial)Group 1 - (Decision Trial - Directed Trial)Group 2]. Cluster-level family-wise error correction (pcorr < 0.05).

^b^If bilateral, the larger maximum is shown.

^c^Total voxels in each cluster. A structure with a number here, and the following structures without numbers, comprise one cluster.

^d^Maximally activated voxel in region.

**Table 5:** The Groups' Activity Differences before Risky Responses.

**All Males > All Females (Excluding Regions of Interaction)** ^a^								**All Cmp > All Pts (Excluding Regions of Interaction)** ^a,b^							
**Structure**	**BA or**	**Cluster**	**x**	**y**	**z**	**t** ^d^	**Region or**	**Structure**	**BA or**	**Cluster**	**x**	**y**	**z**	**t** ^d^	**Region or**
	**Side** ^b^	**Size** ^c^					**Function**		**Side** ^b^	**Size** ^c^					**Function**
Sup Fr Gy	R10	115	22	46	24	3.9	Fr Pole	Inf Fr Gy	L47	243	-38	14	-16	4.4	vlPFC
Mid Fr Gy	L47	697	-34	38	-4	4.0	vlPFC	Insula	L13		-34	12	-16	3.3	Insula
Inf Fr Gy	L47		-34	32	-1	7.8	vlPFC	Sup Temp Gy	L38		-46	16	-20	3.1	Temp
Inf Fr Gy	R47	295	34	32	0	3.7	vlPFC	Mid Temp Gy	R21	198	50	-22	-12	4.3	Temp
Ant Cing Gy	L24,32	778	-14	32	28	5.5	mPFC	Mid Temp Gy	R22	164	52	-36	4	3.3	Temp
Med Fr Gy	L8,32,6,9		-14	30	38	4.2	m & dlPFC	Sup Temp Gy	R22		54	-42	6	3.0	Temp
Cing Gy	L32		-12	22	36	2.8	mPFC	Supramarg Gy	R40	273	58	-50	26	4.0	Par
Sup Fr Gy	L6		-14	20	50	2.8	SMA	Inf Par Lob	R40		46	-50	42	2.8	mPar
**Total**		**1885**						Mid Occ Gy	R18	184	32	-64	0	3.7	Occ
								Cuneus	R17		26	-84	8	2.7	Occ
								**Total**		**1062**					
**All Females > All Males (Excluding Regions of Interaction)** ^a,b^								**Regions Showing Gender X Group Interactions**							
**Structure**	**BA or**	**Cluster**	**x**	**y**	**z**	**t** ^d^	**Region or**	**Structure**	**BA or**	**Cluster**	**x**	**y**	**z**	**t** ^d^	**Region or**
	**Side** ^b^	**Size** ^c^					**Function**		**Side** ^b^	**Size** ^c^					**Function**
Sup Fr Gy	L8	164	-16	46	52	4.7	dlPFC	Med Fr Gy	R10	115	20	56	8	3.7	Fr Pole
Sup Fr Gy	R6,8	901	12	16	54	6.0	SMA, mPFC	Sup Fr Gy	R10		20	50	18	2.7	Fr Pole
Med Fr Gy	R6,8,9,32		10	12	50	3.3	m & dlPFC	Lingual	R	832	2	-48	-24	3.1	Cerebell
Cing Gy	R24,32		16	22	36	3.4	mPFC	Culmen	R		10	-52	-22	3.1	Cerebell
Mid Fr Gy	R6		24	14	58	2.8	SMA	Fastigium	R		10	-54	-28	3.2	Cerebell
Insula	L13	291	-38	12	-10	4.1	Insula	Dentate	R		14	-58	-32	3.2	Cerebell
Inf Fr Gy	L47		-28	16	-16	3.3	vlPFC	Tonsil	R		8	-60	-40	3.4	Cerebell
Claustrum	L		-28	18	0	3.1	Sens Integ	Declive	R		8	-62	-22	3.1	Cerebell
Lingual Gy	L19	113	-14	-48	-4	3.9	Occip	Nodule	R		8	-64	-34	4.7	Cerebell
Parahip Gy	L30		-16	-46	0	3.1	Temp	Pyramis	R		24	-66	-38	3.0	Cerebell
Culmen	L		-12	-46	-6	3.5	Cerebell	Uvula	R		6	-66	-38	3.1	Cerebell
Cing Gy	L31	180	-6	-50	28	3.5	mPar	**Total**		947					
Precuneus	L31		-10	-54	32	2.8	mPar								
Post Cing	L23,30		-4	-50	22	2.7	mPar								
**Total**		**1649**													

Abbreviations, Footnotes: As in Table 4.

### Processing Differences: Males and Females

Before cautious responses males' activity exceeded females' in 14 clusters and broad regions (Table 4, All Males > All Females). The largest cluster, about 45 percent of these voxels, included portions of ventrolateral and medial PFC, striatum, insula, thalamus, brain stem, and other regions. Meanwhile, females' activity exceeded males' in just one cluster that barely exceeded our 97-voxel extent threshold (Table 4, All Females > All Males; Fig 3, green pointer).

Before risky responses males exceeded females, and females exceeded males, in about equally extensive regions. In both cases the largest cluster involved anterior cingulate and medial frontal gyri, with boy-greater activation on the left and girl-greater activation on the right. In addition, medial parietal, temporal, occipital, and cerebellar regions showed only girl-greater activation (Table 5, All Males > All Females; All Females > All Males).

### Processing Differences: Male:Female x Patient:Comparison Interactions

**Fig 4:**
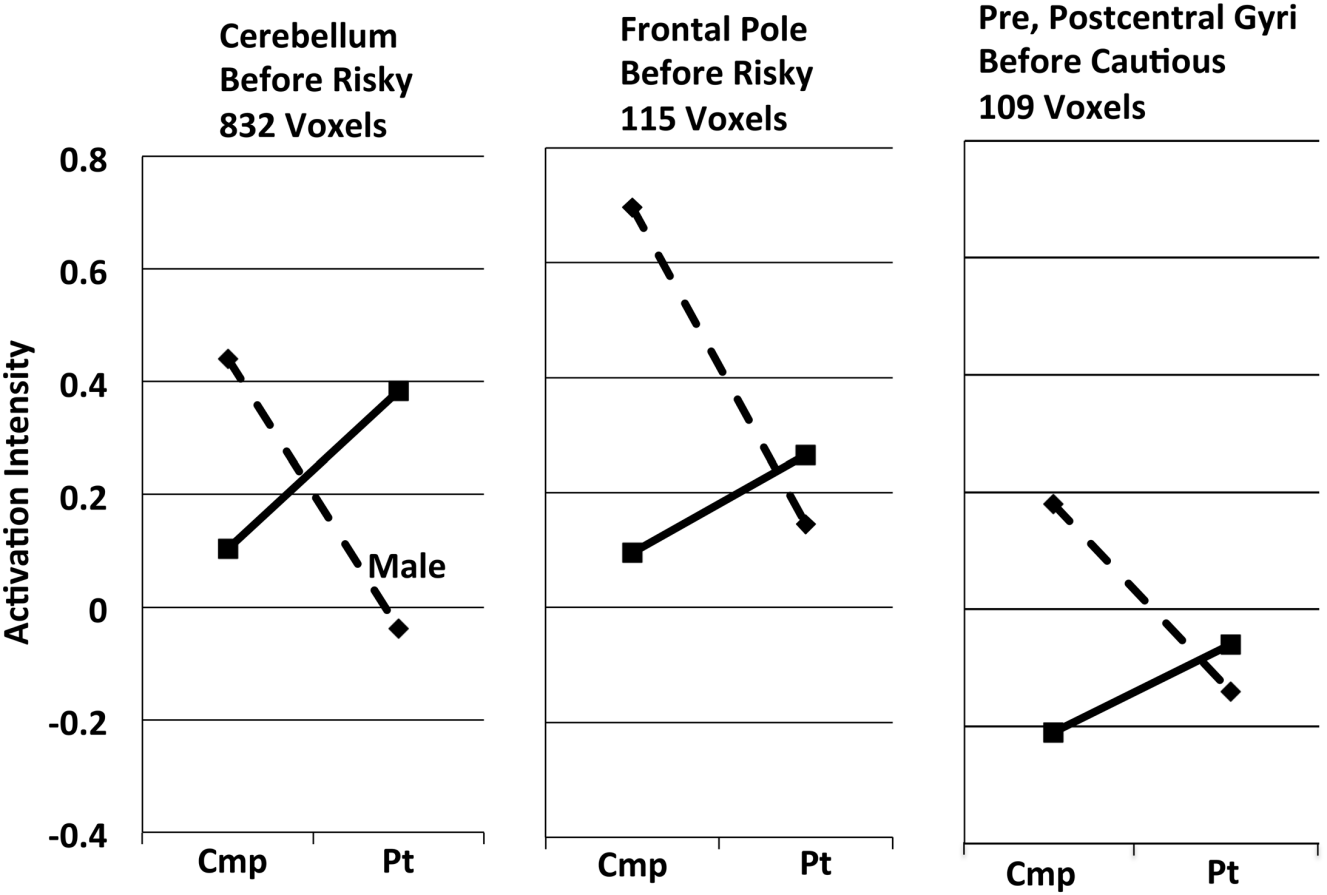
Three brain regions showing significant male:female x comparison:patient group interactions (*cf*., Tables 4 and 5). Dashed lines, males. Solid lines, females. Cmp, comparison participants. Pt, patients. Activation intensity is the mean activation difference (Decision Trial – Directed Trial) for all voxels in the cluster, so negative values indicate relative deactivation in Decision Trials, compared to Directed Trials. Standard errors of the means for each of these points lie in the narrow range ± 0.064–0.094.

Gender x group interactions appeared in only 3 sites (Tables 4 and 5, Regions Showing Gender X Group Interactions; Fig 4). They included a sizable cerebellar cluster and a smaller frontal one before risky responses, and one small cluster before cautious responses.

### Potential Confounds

**Fig 5:**
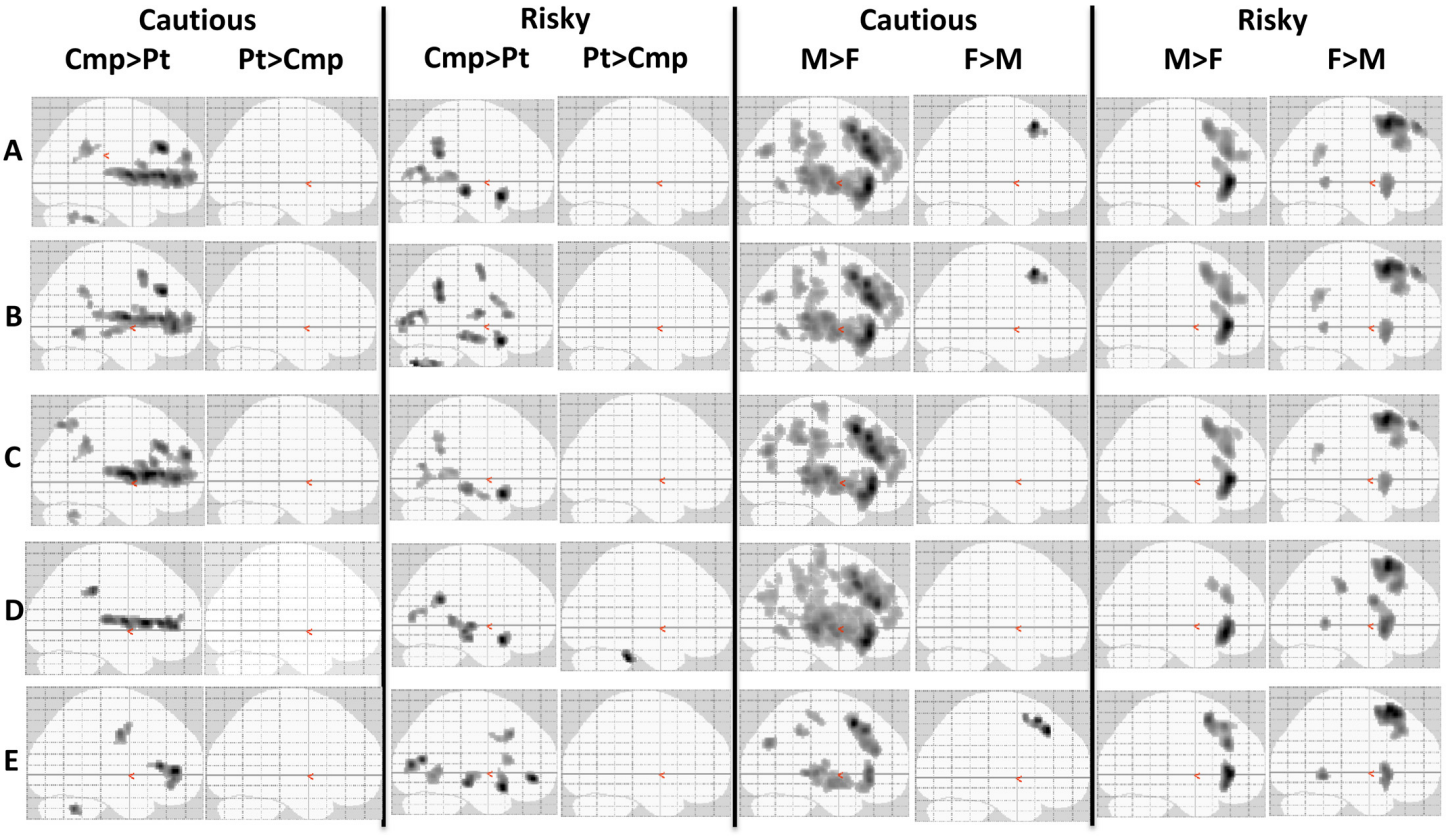
Assessing possible confounds by changing samples or procedures. Clusters activating significantly differently in groups appear as shadows on a 2-dimensional surface; activations preceding cautious or risky behaviors are shown separately. Abbreviations: *Cmp*, comparison participants. *F*, females. *M*, males. *Pt*, patients. Row A: entire sample, data analyzed as in Tables 4 and 5. Row B: same sample, additional adjustment for depression severity (Carroll Rating Scale). Rows C-E: analyses as in Row A, but excluding: left-handed participants (Row C: 2 patient males, 1 comparison male, 3 patient females, no comparison females); or participants receiving psychotropic medications (Row D: see Table 1); or recent regular tobacco smokers (Row E: 6 patient males, 1 comparison male, 20 patient females, 1 comparison female). All images modified identically to increase contrast between the white brain figure and its gray background.

"Glass brain" images condense complex data, allowing visual comparisons of activation patterns. Fig 5, Row A, shows group main effects with activation intensity adjusted for age and IQ, as in Tables 4 and 5. Before both cautious and risky behaviors comparison participants activated many regions significantly more than patients, and patients activated none more than comparison adolescents. Before cautious behaviors boys' activity significantly exceeded girls' in many regions, while girls' activity exceeded boys' in one 99-voxel cluster; before risky behaviors each sex activated some regions more than the other did.

Fig 5, Row B additionally adjusts for depression severity, Row C excludes left-handed subjects, and Row D excludes participants taking prescribed psychotropic medications. These manipulations only modestly changed the patterns seen in Row A. We conclude that variations in depression severity, or inclusion of left-handed or medicated participants, had little effect on the strong activation differences of patients vs. comparison youths, or of males vs. females.

Row E excludes recent regular smokers. Although some similarities with Row A remain (*e*.*g*., patients activate no structures more than comparison youths), Row E is severely compromised because the probability of recent regular smoking excluded 20 of 21 female patients, and 6 of 20 male patients, leaving only 15 patients for that contrast. Thus, our data cannot rule out an effect of smoking on those activation differences. Recognizing this, future studies might utilize non-categorical smoking measures, such as cotinine or carbon monoxide levels, as covariates in analyses of fMRI activation intensity.

## Discussion

Adolescent risk-taking too often leads to injury or death from drug overdoses, accidents, or homicides. Individual youths with behavioral disinhibition, and boys generally, are especially vulnerable to these tragedies. We modeled adolescents' frequent choosing between doing a risky behavior, or its cautious alternative. We show that adolescents' pre-response neural processing during decision-making differed in those with or without severe behavioral disinhibition, and differed between boys and girls.

We sought regions in which groups generated different activation while making decisions, examining activation differences occurring during the 4-sec deliberation preceding responses, using this contrast:

$${\left(\text{Decision Trial – Directed Trial}\right)}_{Group\;A}–\;{\left(\text{Decision Trial – Directed Trial}\right)}_{Group\;B}$$

Thus, our tables include only regions activating differently in the groups. Of regions doing so, many activated before both risky and cautious behaviors. Recent reviews [47,54] suggest that these regions are active before both behaviors because they are the "decision makers" that choose to "go risky" or "go cautious". Other regions activated before just one of the behaviors; we consider them as "choice implementers", sustaining an already-made decision (of one type) and preparing the youth for its outcome.

### Patient and Comparison Youths Differently Process Risky-or-Cautious Decisions

Our patients had strong behavioral disinhibition with associated real-life high-risk behaviors (Fig 1b; Table 2). Before *risky behaviors* comparison participants activated 5 clusters more than patients (Table 5, All Cmp > All Pts), and patients activated no regions more than comparison youths. Patients showed hypoactivity in numerous regions critically contributing to decision-making, including the following:

Insula contributes to the selection of behavioral choices by providing information about internal states (emotions, physical sensations, etc.) [55]. It also assesses the risk of aversive outcomes from those choices and monitors the accuracy of those risk predictions [56].

Patients also showed hypoactivity in right posterior parietal cortex (PPC, BA 40), as well as bilateral frontal poles (BA 10; Table 5, All Cmp > All Pts). Frontoparietal circuits are part of "a core reward network" [57] for risky-decision processing; "it is crucial for the parietal lobule to be involved in the anticipation stage of reward processing so as to plan and prepare for an informed action" [58]. PPC also contributes to attention and cognition [59] and generates intentions to move [60].

Together, PPC and middle temporal gyrus store, update, and make available internal representations of reward-punishment contingencies, functions highly relevant to BD's risk-taking. First, middle temporal gyrus (BA 21) and other regions store memories thought to be "rules" (response contingencies) for one’s ongoing activity [61]. BA 21 is activated by inferior frontal gyrus (BA 47), which itself is activated [62] by task-salient external cues (*e*.*g*., our task's stoplights). So when BA 47 “sees” those cues, it apparently retrieves the task's contingency rules from middle temporal gyrus (BA 21) and elsewhere [63]. Additionally, as BA 47 responds to environmental cues, PPC (BA 40) co-activates, the two working together to repeatedly revise representations of changing environmental contingencies [62].

In our game all of those regions were hypoactive in patients before risky behaviors, and we propose that such hypoactivity contributes to patients' disastrous real-life decisions (Table 2). Others propose that persons with substance problems may engage in disinhibited reward seeking because of either overactive [64] or underactive [65] dopaminergic "reward" systems. Although within-group analyses generally are beyond the scope of this report, those analyses (*e*.*g*., S1 Fig) do show robust activation (presumably, reward anticipation) in dopaminergic structures before risky responses; however, before those responses we see no significant, overall patient-comparison differences there (Table 5). Thus, our data suggest that while decisions are being made, patient and comparison participants' activations differ in systems of inhibitory control, rather than reward.

During deliberation before *cautious responses* patients activated no regions more than comparison youths, but comparison youths (more than patients) activated one very large, and three smaller clusters (Table 4, All Cmp > All Pts). They included:

Frontal pole (BA 10), where boys with conduct disorder reportedly have reduced gray-matter volume [29], activated more in comparison youths. Critical for behavioral flexibility, frontal pole tracks the relative advantage of switching among behaviors, thereby influencing response-choosing; lesions there severely impair the open-ended, little-structured decision-making of everyday life [66]. Impaired behavior-switching with perseveration in previously-reinforced but now-punished behaviors, characterizes psychopathic adults [46]. BA 10 integrates information needed for selecting responses, and it robustly activates when subjects defer one task to do another. Crucially, individual differences in BA 10 activation predict differences in effectively adapting behavior [66].

Medial prefrontal cortex (mPFC) includes anterior cingulate and medial frontal gyri (BA 25, 32). The mPFC monitors which actions are earning rewards or punishments, and with what probability. It integrates that information with other incoming information before signaling other brain areas to adjust behavior for maximizing reward [67].

Cerebellar regions were hypoactive in patients before cautious responses (Table 4, All Cmp > All Pts). Cerebellum's role in cognition involves cortico-cerebellar circuits to temporal and posterior parietal cortices [68], regions also hypoactive in these patients. Lesions of the "cognitive cerebellum" (Crus I and II) produce a "cerebellar cognitive affective syndrome" with disinhibition, impulsivity, inattentiveness, distractability, hyperactivity, anger, and aggression [68,69]. Many of those symptoms appear in our patients, who also have a deficiency of cerebellar gray matter [27]. Moreover, infants and toddlers at familial risk for alcohol problems and other BD-like behaviors also show gray-matter abnormalities in cerebellum, accompanied by delayed onset of walking and sitting, reduced muscle tone, excessive body sway, and impaired control of ocular saccades [70]. Similarly, by 11–15 years of age such high-risk youngsters, despite not yet using substances, have reduced functional and structural connectivity in fronto-cerebellar circuits [71]. Our findings support suggestions that cerebello-frontal circuits contribute to the pathology of BD.

PPC also was less active in patients than in comparison youths before cautious responses. As noted above, PPC (BA 40) maintains representations of task contingencies and contributes to attention, cognition, and intentions to move [59,60]. Finally, comparison participants more than patients activated striatal structures before cautious behaviors.

#### Neural Influences on Patients' Behavior

Patients' widespread neural hypoactivity during risky-or-cautious decision-making suggests a severe biological impairment that contributes to their profound, real-life substance and antisocial problems. Sustaining sincerely intended cautious decisions, *e*.*g*., to remain abstinent, is very difficult for youths with conduct and substance problems. Hypoactivity in numerous control structures may permit environmental circumstances (*e*.*g*., peer pressure, or the availability of drugs or sex partners) to more easily shift such patients' decisions from adaptive cautious behaviors toward dangerous risky ones.

Non-invasive direct-current stimulation at the scalp alters both cortical neuronal activity and risky decision-making [72,73]. The top two rows of Fig 3 map patients' accessible hypoactive cortical regions, posing the question of whether stimulation there could clinically benefit such patients.

Patients like these took more risks in another laboratory task [21]; why did these patients not take more risks than comparison youths in the present game? We suggest four reasons: (*i*) Required pre-response deliberations, like the present 4-sec deliberation, strongly suppress antisocial persons' tendency to continue doing previously reinforced, but currently punished, behaviors [46]. (*ii*) Although our task clearly revealed abnormal neural-control mechanisms in patients, those mechanisms may be adequate for making 90 simple "press left or right" choices, while being inadequate for complex real-life decisions (*e*.*g*., "Shall I stay home to study or sneak out to get drunk?") (*iii*) Adverse win-loss ratios (5-cent win vs. 10-cent loss) may have somewhat suppressed patients' excessive risk-taking. (*iv*) This task's instantaneous rewards and punishments made clear the decreasing probability of reward, perhaps further constraining patients' risk-taking.

Is patients' drug use the source of their neural impairment in decision-making? They had complex differing severities of, *e*.*g*., conduct disorder, ADHD, and substance use disorders involving tobacco, alcohol, cannabis, and other drugs. Moreover, drug exposure does alter brain function (*e*.*g*., [74–76]) and possibly structure [77]. However, BD is assessed as the considerable comorbidity among such externalizing disorders, and it reflects shared genetic influences on them. Thus, we chose not to "clean" the sample by eliminating youths with such "confounds"; the "confounds" in fact manifest and quantify the poor judgment, impulsivity, impaired self-control, and risk-taking that comprise behavioral disinhibition. BD's severity is reflected in the number and severity of those inherently related conditions, and a "cleaned" sample by definition has less severe BD.

But then, can an underlying neural signal from BD "shine through" the various neural signals of past substance use and other disorders, all with differing severity? In fact, the literature suggests that the serious substance and antisocial problems characterizing BD are strongly and persistently associated with neural hypoactivity in decision-related structures—before, as well as after—substance exposure. To wit, (*i*) hypoactive P300 responses in prepubertal children without substance exposure *predict* later conduct and substance problems [16]. (*ii*) Among 12–14 year-old youngsters with little drug exposure, widespread brain hypoactivity on an inhibitory task *predicts* later substance and conduct problems [35]; that hypoactivity included several regions that were hypoactive in our patients. (*iii*) Youths 13–15 years old with little alcohol exposure but at familial risk for alcoholism show brain hypoactivity in a risky-decision task [42]. (*iv*) Among 14 year-old adolescents with little drug exposure, greater risk-taking is associated with reward-system hypoactivity [36]. (*v*) Even among adults hypoactivity of decision-making structures predicts disinhibited behavior, such as relapse after substance treatment [78] or recidivism after prison release [79]. Varied disorders, tobacco smoking, and exposure to therapeutic and illicit drugs probably did influence our results, but brain hypoactivity like that reported here is found in substance-free youngsters *at risk* for conduct and substance problems. We extend those observations to adolescents who now have those problems.

Finally, some [80] suggest that fMRI comparisons require groups of at least 20. We know of no previous fMRI studies of adolescents with serious substance and conduct problems that compared male and female samples of that size, and excluding tobacco smokers would have excluded nearly all of our female patients. Understanding these disorders requires studying youths with comorbidity.

#### Comparison:Patient x Male:Female Interactions

Comparison participants activated all of the regions discussed above more than patients (Tables 4 and 5, All Cmp > All Pts). Males followed that pattern even in the three regions with sex x group interactions (Fig 4), but females reversed it there; patient females activated more than comparison females. We noted above a role in decision-making for cerebello-frontal circuits, and the large cerebellar interaction cluster (Fig 4) may suggest that during decision-making, hypoactive brains of females, but not males, compensate by more vigorously activating cerebello-frontal circuits.

### Boys and Girls Differently Process Risky-or-Cautious Decisions

Among humans [2] and many other animals [8] males more than females engage in aggressive, dangerous risk-taking. Although all groups significantly reduced their risky responding as losses increased (Fig 2), girls overall (compared to boys) made fewer risky and more cautious responses, thereby experiencing fewer risky wins and losses. Comparison girls' risky responses came more slowly than comparison boys', and girls also failed at timely responding much more than boys (Table 3). Thus, despite many similarities to boys, girls behaved differently. Moreover, that behavioral difference was preceded by large differences in the neural processing of decision-making, a strong sexual dimorphism of brain function that was linked to sex differences in risky behaviors.

One gender difference was that before *risky responses* boys more than girls activated *left* medial PFC (BA 24 and 32, along with both medial and dorsolateral BA 9), while girls activated the corresponding *right* structures more than boys (Table 5, All Males > All Females; All Females > All Males). Also, the rostral inferior frontal gyrus (BA 47) activated bilaterally more in boys than girls, especially on the left (*t* = 7.8, the highest observed here), although a more caudal region of left BA 47 did activate more in girls. Numerous other regions (Table 5, All Females > All Males), most rather caudal, and several cerebellar, activated more in girls than boys.

The regions activating more in males than females before *cautious responses* comprised by far the largest group difference in this study (Table 4, All Males > All Females). Many decision-making regions discussed above were included there: frontal pole, medial PFC, dorsolateral PFC, insula, inferior frontal gyrus (BA 47), and posterior parietal (BA 40) and orbitofrontal (BA 11) cortices.

Participants in this game frequently switched between risky and cautious choices. Regions involved in adaptive behavior-switching include frontal pole, medial and dorsolateral PFC, and pre-supplementary motor area (BA 6), all of which project to striatum and subthalamic nuclei; the latter structures, apparently after receiving a switch-related signal from pre-supplementary motor area, suppress ongoing (but no-longer advantageous) behavior, allowing implementation of new behaviors [81,82]. All of those structures and circuits were significantly more active in boys than girls before cautious responses.

Only in right mPFC did girls activate more than boys one small cluster (Table 4, All Females > All Males).

#### Neural Influences on Girl-Boy Behavior Differences

The sexes differed, especially as youths made (and sustained for 4 seconds) decisions for cautious behaviors; then, in numerous regions males' activation intensity significantly exceeded females'. Also, males made more risky responses here than females, and in everyday life more males than females take dangerous risks. We therefore suggest: (*i*) in adolescent males risky-or-cautious decision-making circuits (including medial, dorsolateral, and ventrolateral PFC; insula; PPC, and others) are less biased than in females to make cautious decisions; (*ii*) males compensate by massively recruiting auxiliary structures to make and sustain such decisions; and (*iii*) despite that recruitment males still make fewer cautious choices than females.

Finally, we note that important previous studies *e*.*g*., [35–37,39,83] have successfully combined male and female adolescents. However, our finding of strong sex differences suggests that future studies of adolescent risk-taking should compare the sexes before combining them.

### Limitations

Several concerns or criticisms may influence conclusions from this study.

First, although we find strong average sex differences in neural processing of risky-cautious decisions and in risky behavior, the data of course do not argue that all males take more risks, or are less cautious, than all females. Second, few have studied adult sex differences in risky decision-making [*e*.*g*., 13,14]; without more information, our results should not be generalized to adults.

Third, boys had more "wins" in the game than girls (Table 3). Did the brain activity differences of the two groups just result from experiencing different numbers of "wins"? Addressing this, we re-analyzed pre-response brain activity after controlling for each subject's total wins. That resulted in only small changes from the activity patterns of Tables 4 and 5; *e*.*g*., before cautious responses the All Male > All Female voxel count declined only about 1 percent, and females still activated the same small cluster more than males. Similarly, before risky responses the previous patterns persisted with little change. We conclude that "win" experiences were not a major cause of the observed male:female activation differences.

Fourth, inattention is one of the characteristics comprising the BD trait. Did inattention to this task cause patients' neural hypoactivity? Missing responses may indicate inattention, and both male and female patients missed responding significantly more often than same-sex comparison youngsters (Table 3). Also, patient females' inattention ratings were particularly high (Table 2). So inattention, *itself a key aspect of BD*, may have played some role in patients' neural hypoactivity.

Fifth, we clearly show group differences as adolescents process decisions to "go risky" or "go cautious". Our data, however, cannot show whether those differences are unique to risky-or-cautious decision-making, or whether they also extend to other decisions, or to other cognitive processes.

Sixth, patients' mean IQ was significantly lower (≈ 8 points) than comparison participants' (Table 2). It has been known for over 20 years that "One of the most robust findings in the study of antisocial behavior is an IQ deficit of … about 8 IQ points, between juvenile delinquents and their nondelinquent peers" [84]. IQ deficits are a part of the neuropsychology of conduct disorder. Recruiting patient and comparison samples to minimize IQ differences might assure similar cognitive capacity for task performance, but might also minimize group differences on other crucial variables, such as antisocial severity. Thus, we accepted group differences in IQ, but we controlled for IQ (and age) in our analyses. Only further research can clarify the best approach to this issue.

Seventh, did our task function as intended? Decision and Directed Trials were identical in visual and auditory stimuli, but Decision Trials required decisions. Did the (Decision – Directed) contrast actually highlight decision-related neural activity? Our previously unpublished within-group analyses are, for the most part, beyond the scope of this report, but in such analyses decision-related structures (*e*.*g*., striatum in S2 Fig) almost always show no activity in the (Directed Trial – Decision Trial) contrast, and either more activity or no difference in the (Decision Trial – Directed Trial) contrast. On the other hand, structures in the Default Mode network (*e*.*g*., precuneus in S2 Fig), should be less active during decision-making, and they often show more activity in the (Directed Trial – Decision Trial) contrast. Finally, we occasionally find sites where activation during Decision Trials is less than that during Directed Trials; *e*.*g*., the right panel of Fig 4 shows several within-group data points having negative values, and we consider them to reflect actual deactivations below baseline (*i*.*e*., below Directed Trial levels). Thus, the weight of evidence supports the view that this task's fundamental contrast functioned as intended.

Eighth, our basic contrast was (Decision Trial Activation Trial – Directed Trial Activation). However, during Decision Trials cautious responses earned only 1 cent, while correct responses in Directed Trials (the intended baseline control condition) earned 2 cents. Did that larger Directed-Trial reward induce an artifact of unwanted reward activity? Our previously unpublished *within-group* analyses of (Decision Trial Activation – Directed Trial Activation) showed strong reward-anticipating activation in ventral striatum and other dopaminergic "reward" structures during deliberations preceding *risky* responses (*e*.*g*., S2 Fig). Before *cautious* responses striatal activity in that contrast was weaker but still visible; meanwhile, the (Directed Trial Activation Trial – Decision Trial Activation) was consistently less and usually nil (S3 Fig). That is, (*i*) cautious left responses earned one cent in Decision Trials; (*ii*) similar left responses earned two cents in Directed Trials; but (*iii*) before left responses there was considerably more reward-anticipating activation in Decision Trials than in Directed Trials. Although self-selected left responses in Decision-Trials earned less, they still produced greater reward anticipation than left responses in Directed Trials, so our baseline Directed Trials did function as intended.

Ninth, unlike just looking at a fixation screen, our Directed Trials were an active control condition, sharing with Decision Trials identical sights, sounds, and motor movements, as well as similar rewards. However, Decision Trials additionally required a risky or a cautious decision, so that the difference between the trial types could isolate decision-related activation. Active control conditions are widely used in fMRI studies, but if their stimuli induce, *e*.*g*., unrecognized fear or anger, neural-activation artifacts could result. Fortunately, although such artifacts can never be completely ruled out, they are very unlikely here because of the extreme similarity of the active control and the Decision Trial.

Tenth, we did not evaluate callous-unemotional traits, so we could not test a valuable recent proposal that those traits distinguish two types of conduct disorder with separate neuropathologies [34]. That proposal also emphasized a role for amygdala in those pathologies. However, although our risky-or-cautious task produced very different brain activation in patients and comparison youths, amygdala did not activate differently. Moreover, we found strong sex differences in the processing of risky-or-cautious decisions, but that proposal [34] does not consider sex differences. Discrepancies between that useful formulation and our findings emphasize a need for continued research on the neuropathology of these disorders.

## Implications and Future Research

First, adolescents with serious conduct and substance problems have life-threatening deficits in risky-or-cautious decision-making, and Fig 3 maps hypoactive cortical regions as such youths make those decisions. Direct-current brain stimulation at the scalp influences neuronal activity and risky decision-making [72,73], and our maps could guide stimulation researchers seeking improved treatments for those patients.

Second, recognition of sex differences in the neural processing of risky-or-cautious decisions may help researchers improve psycho-educational interventions, *e*.*g*., [85], to better guide boys toward more cautious, less dangerous choices, aiming to reduce their excess mortality from accidents, homicides, and overdoses.

Finally, the United States incarcerates proportionately more juveniles than any other nation [86], although the United States Supreme Court ruled that, because of immature neural development, adolescents generally have diminished culpability for illegal acts [87]. We show that, beyond adolescents generally, those with strong behavioral disinhibition have additional brain impairments during risky-or-cautious decision-making, decision-making that may end in juvenile-justice involvement. This suggests that during sentencing of adolescent offenders, evidence of heritable behavioral disinhibition might be viewed as further reducing criminal culpability, perhaps tipping dispositions less toward punitive and demonstrably harmful incarcerations [86,88], and more toward probation with treatment.

## Supporting Information

S1 Fig Four groups' within-group reward-anticipating striatal activation before *risky* responses. Family-wise error correction at voxel level, *p*_*corr*_ = 0.05, y = 3. Contrast: Decision Trials – Directed Trials. In each image the range of significant *t* values = 4.95 (minimum) to 10 or 11 (maximum).
(TIF)

S2 Fig Within-group activations in opposite contrasts during 4 sec deliberation periods before *risky* responses. This example: images from control boys. Analysis: Cluster-level family-wise error correction, extent threshold, 97 voxels at *p*_*uncorr*_ = 0.005; *p*_*corr*_ = 0.05. For each row the minimum significant *t* = 4.95; color bars show the maxima. *R*, right. With the mental effort of pre-response decision-making ventral striatum activated more in Decision Trials than in the no-decision Directed Trials (red arrow). Conversely, as part of the Default Mode network, which activates more when mental effort is reduced, precuneus activated more in Directed Trials than in Decision Trials (yellow arrow).
(TIF)

S3 Fig Within-group activations in opposite contrasts during 4 sec deliberation periods before *cautious* responses. Images from all 4 groups at *y* = 8 (note that S1 Fig was cut at y = 3). Analysis: as in S2 Fig. At colored regions the designated contrast is significantly greater than zero; minimum significant t-value in all images = 4.95. Green arrow, ventral striatum. *Caut*, Cautious responses; *Dec*, Decision Trials; *Dir*, Directed Trials; *R*, right.
(TIF)
