# Supplementary figures and images for: Adolescents' Neural Processing of Risky Decisions: Effects of Sex and Behavioral Disinhibition
Source: PLoS One. 2015 Jul 15;10(7):e0132322. doi: 10.1371/journal.pone.0132322 (PMC4503769; doi:10.1371/journal.pone.0132322)

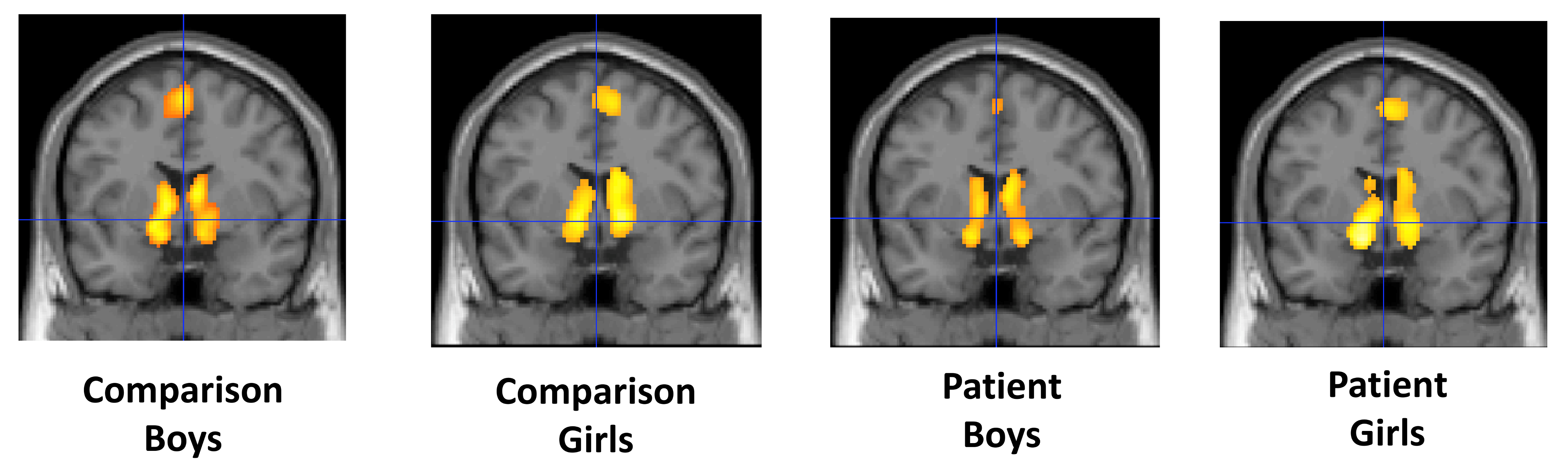

Supplement: S1 Fig — Family-wise error correction at voxel level, p corr = 0.05, y = 3. Contrast: Decision Trials – Directed Trials. In each image the range of significant t values = 4.95 (minimum) to 10 or 11 (maximum). (TIF) [file pone.0132322.s001.tif]

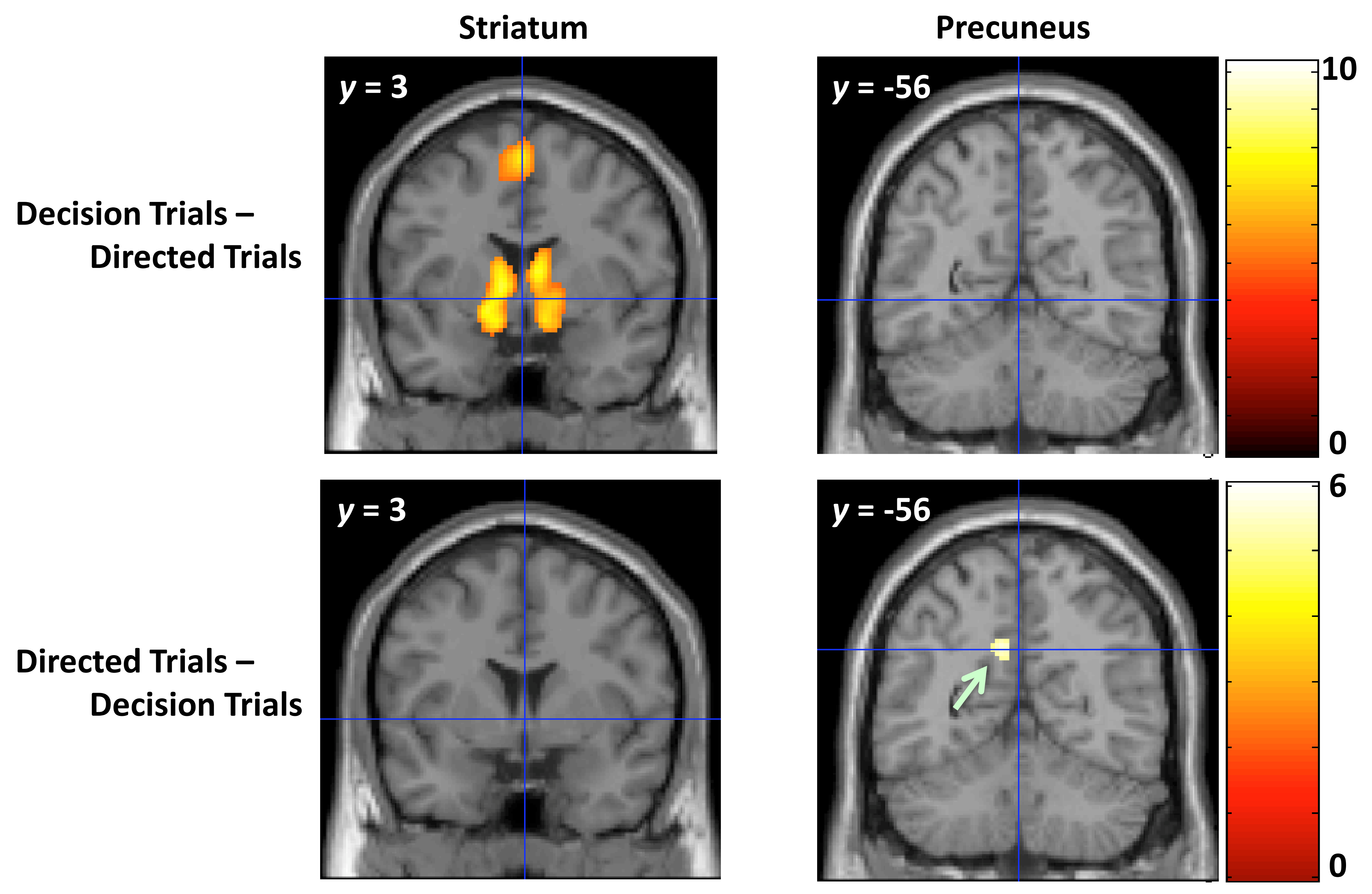

Supplement: S2 Fig — This example: images from control boys. Analysis: Cluster-level family-wise error correction, extent threshold, 97 voxels at p uncorr = 0.005; p corr = 0.05. For each row the minimum significant t = 4.95; color bars show the maxima. R, right. With the mental effort of pre-response decision-making ventral striatum activated more in Decision Trials than in the no-decision Directed Trials (red arrow). Conversely, as part of the Default Mode network, which activates more when mental effort is reduced, precuneus activated more in Directed Trials than in Decision Trials (yellow arrow). (TIF) [file pone.0132322.s002.tif]

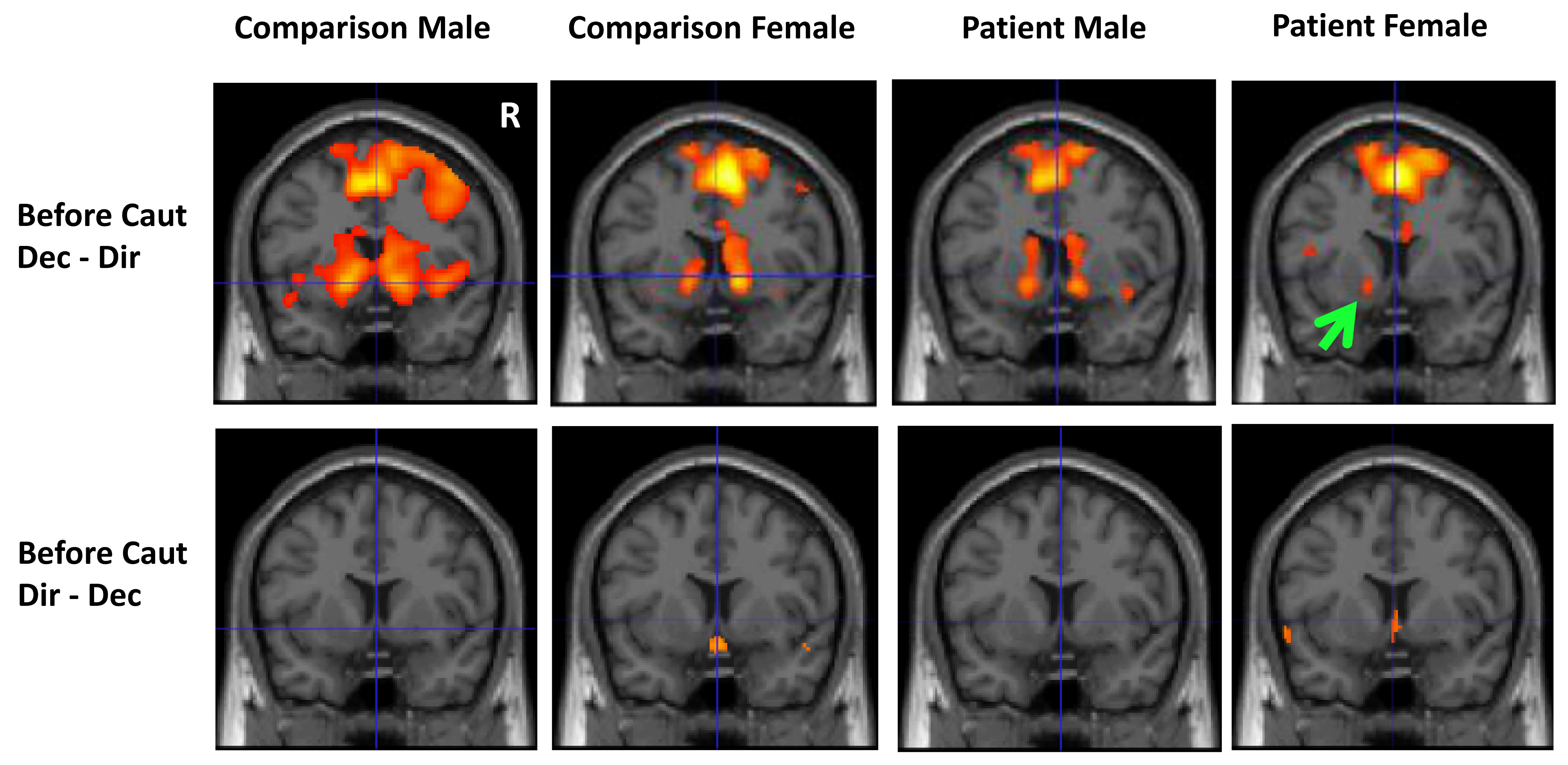

Supplement: S3 Fig — Images from all 4 groups at y = 8 (note that S1 Fig was cut at y = 3). Analysis: as in S2 Fig. At colored regions the designated contrast is significantly greater than zero; minimum significant t-value in all images = 4.95. Green arrow, ventral striatum. Caut, Cautious responses; Dec, Decision Trials; Dir, Directed Trials; R, right. (TIF) [file pone.0132322.s003.tif]
